# Supplementary material for: Association of Alternative Markers of Carbohydrate Metabolism (Fructosamine and 1,5-Anhydroglucitol) with Perioperative Characteristics and In-Hospital Complications of Coronary Artery Bypass Grafting in Patients with Type 2 Diabetes Mellitus, Prediabetes, and Normoglycemia
Source: Diagnostics (Basel). 2023 Mar 3;13(5):969. doi: 10.3390/diagnostics13050969 (PMC10000986; doi:10.3390/diagnostics13050969)
Supplement: Supplementary file 1 [file diagnostics-13-00969-s001.zip › diagnostics-2233923-supplementary.pdf]

Supplementary Table S1. Perioperative characteristics of patients in groups with composite endpoint-1 (significant perioperative complications + extended hospital stay after surgery) and without it.

| Signs | Group 1 With combined endpoint n = 291 | Group 2 Without combined endpoint n = 92 | p |
|---|---|---|---|
| Anamnestic and clinical characteristics | | | |
| Men / women (n, %) | 205/86 (70.5/29.5) | 79/13(85.5/14.1) | 0.003 |
| Age (years, Me [ LQ ; UQ ]) | 60.0 [55.0; 60.0] | 57.0 [51.0; 60.5] | <0.001 |
| Any disorders of CMD (n, %) | 156 (53.6) | 36 (39.1) | 0.015 |
| Type 2 diabetes (n, %) | 109 (37.5) | 16 (17.4) | <0.001 |
| Prediabetes (IFG, IGT) (n, %) | 47 (16.2) | 20 (21.7) | 0.002 |
| Normoglycemia (n, %) | 135 (46.4) | 56 (60.9) | 0.002 |
| BMI ( kg/m $^2$ , Me [LQ; UQ]) | 29.1 [25.9; 32.2] | 27.0 [24.1; 30.0] | <0.001 |
| Obesity (BMI ≥30 kg / m $^2$, n, %) | 125 (42.9) | 24 (26.1) | <0.001 |
| Arterial hypertension (n, %) | 264 (90.7) | 79 (85.9) | 0.185 |
| Angina class III - IV (n, %) | 113 (38.8) | 34 (37.0) | 0.591 |
| Heart failure class NYHA III-IV (n, %) | 84 (28.9) | 18 (19.6) | 0.172 |
| Smoking (n, %) | 86 (29.6) | 42 (45.7) | 0.004 |
| Myocardial infarction history (n, %) | 182 (62.5) | 58 (63.0) | 0.668 |
| Stroke history (n, %) | 21 (7.2) | 6 (6.5) | 0.820 |
| Previous PCI (n, %) | 26 (8.9) | 11 (12.0) | 0.392 |
| Previous CABG (n, %) | 82 (0.7) | 2 (2.2) | 0.797 |
| Carotid surgery (n, %) | 8 (2.8) | 3 (3.3) | 0.803 |
| Limb arteries surgery or amputation (n, %) | 2 (0.7) | 1 (1.1) | 0.704 |
| EuroSCORE II (%, Me [LQ; UQ]) | 1.34 [1.23; 2.91] | 1.32 [0.88; 2.10] | 0.002 |
| EuroSCORE II (points, Me [LQ; UQ]) | 2.0 [1.0; 3.0] | 2.0 [1.0; 3.0] | <0.001 |
| CABG characteristics | | | |
| Cardiopulmonary bypass (n, %) | 268 (92.1) | 78 (84.8) | 0.038 |
| Isolated coronary artery bypass grafting (n, %) | 263 (90.4) | 91 (98.9) | 0.007 |
| Combined surgery (n, %) | 28 (9.6) | 1 (1.1) | 0.007 |
| • Carotid endarterectomy (n, %) | 8 (8.2) | 0 (0) | 0.108 |
| • Ventriculoplasty (n, %) | 13 (4.5) | 1 (1.1) | 0.132 |
| • Radiofrequency ablation (n, %) | 14 (4.8) | 0 (0) | 0.032 |
| • Mitral valve (n, %) | 1 (0.3) | 0 (0) | 0.573 |
| • Aortic valve (n, %) | 3 (1.0) | 0 (0) | 0.328 |
| Cardiopulmonary bypass duration (minutes, Me [LQ; UQ]) | 98.0 [81.0; 116.0] | 86.5 [73.0; 103.0] | 0.002 |
| Aortic clamping time (minutes, Me [ LQ; UQ ]) | 63.0 [50.0; 75.0] | 60.0 [49.0; 72.0] | 0.331 |
| Total duration of surgery (minutes, Me [ LQ; UQ ]) | 246 [210.0; 298.0] | 210.0 [195.0; 264.0] | 0.006 |
| Number of shunts (Me [ LQ ; UQ ]) | 3.0 [2.0; 3.0] | 2.0 [2.0; 3.0] | 0.352 |
| Number of distal anastomoses (Me [ LQ; UQ ]) | 3.0 [2.0; 3.0] | 2.0 [2.0; 3.0] | <0.001 |
| Preoperative fasting blood laboratory parameters (Me [LQ; UQ]). | | | |
| GFR CKD-EPI (ml/min/1.73m $^2$ ) | 80.4 [64.0; 98.6] | 81.4[63.2; 98.6] | 0.906 |
| HDL cholesterol (mmol/l ) | 1.0 [0.8; 1.2] | 1.0 [0.8; 1.2] | 0.286 |

| | | | |
|---|---|---|---|
| LDL cholesterol (mmol/l ) | 2.9 [2.2; 3.7] | 3.0 [2.3; 3.9] | 0.304 |
| Triglycerides (mmol/l ) | 2.0 [1.4; 2.5] | 1.6 [1.2; 2.2] | 0.046 |
| HbA1c (%) | 5.5 [5.1; 7.1] | 5.3 [5.0; 5.6] | 0,009 |
| Glucose, venous plasma (mmol/l ) | 5.8 [5.2; 6.9] | 5.5 [5.1; 6.2] | 0.031 |
| Fructosamine (μmol/l) | 286.0 [252.0; 320.0] | 264.5 [201.0; 298.0] | <0.001 |
| 1.5 anhydroglucitol (mcg/ml) | 21,3 [16,5; 25.2] | 20.5 [17.8; 26.0] | 0,483 |
| Preoperative echocardiogram (Me [LQ; UQ]) | | | |
| LV end-diastolic volume (ml) | 156.0 [132.0; 191.0] | 150.0 [129.0; 172.0] | 0.125 |
| LV end-diastolic dimension (cm) | 5.6 [5.2; 6.2] | 5.5 [5.1; 6.0] | 0.051 |
| LV end-systolic volume (ml) | 63.0 [47.0; 97.0] | 62.2 [48.0; 96.5] | 0.421 |
| LV end-systolic dimension (cm) | 3.8 [3.4; 4.7] | 3.8 [3.3; 4.6] | 0.467 |
| Left atrium (cm) | 4.3 [4.0; 4.5] | 4.2 [3.8; 4.4] | <0.001 |
| LV ejection fraction (%) | 60.0 [50.0;64.0] | 61.0 [50.0; 64.0] | 0.958 |
| LV myocardial mass by Deveraux and Reichek (g) | 304.3 [250.5; 375.0] | 242.1 [276.0; 333.7] | 0.010 |
| LV myocardial mass index (g/m$^2$ ) | 159.2 [133.5; 192.0] | 150.3 [124.2; 175.0] | 0.015 |
| Coronary angiography data | | | |
| 1-vessel disease * | 64 (22.0) | 16 (17.4) | 0.343 |
| 2-vessel disease * | 88 (30.2) | 25 (27.2) | 0.274 |
| 3-vessel disease * | 121 (41.6) | 45 (48.9) | 0.216 |
| Left Main Coronary Artery Stenosis >50% | 61 (21.0) | 24 (26.1) | 0.302 |

***Notes:*** *Me [LQ; UQ] - median with upper and lower quartile, CMD - carbohydrate metabolism disorders, IFG - impaired fasting glucose, IGT - impaired glucose tolerance, BMI - body mass index, FC - functional class, NYHA - New York Heart Association, PCI - percutaneous coronary intervention, LOS - length of stay, CABG - coronary artery bypass grafting, EuroSCORE II – European System for Cardiac Operative Risk Evaluation, CMD - carbohydrate metabolism disorders, HDL - high-density lipoprotein, LDL - low-density lipoprotein, GFR - glomerular filtration rate, CKD-EPI - Chronic Kidney Disease Epidemiology Collaboration, LV- left ventricle.*

Supplementary Table S2: Receiver operating characteristic curve analysis. Performance of carbohydrate metabolism preoperative parameters in discriminating the risk of the composite endpoint-1 development (significant perioperative complications + extended hospital stay after surgery) after CABG. Area under the curve

| Test Result Variable(s) | Area | Std. Error[a] | Asymptotic Sig.[b] | Asymptotic 95% Confidence Interval | |
|---|---|---|---|---|---|
| | | | | Lower Bound | Upper Bound |
| Fructosamine before surgery | 0.629 | 0.035 | 0.001 | 0.560 | 0.698 |
| Glucose before surgery | 0.563 | 0.037 | 0.093 | 0.490 | 0.635 |
| Disorders of carbohydrate metabolism | 0.534 | 0.036 | 0.366 | 0.463 | 0.604 |
| Diabetes mellitus | 0.586 | 0.035 | 0.021 | 0.518 | 0.654 |
| Impaired fasting glycemia | 0.498 | 0.037 | 0.959 | 0.425 | 0.571 |

a. Under the nonparametric assumption

b. Null hypothesis: true area = 0.5
